# New bipyridine gold(III) dithiocarbamate-containing complexes exerted a potent anticancer activity against cisplatin-resistant cancer cells independent of p53 status

**DOI:** 10.18632/oncotarget.13448

**Published:** 2016-11-18

**Authors:** Muhammad Altaf, Muhammad Monim-ul-Mehboob, Abdel-Nasser Kawde, Giuseppe Corona, Roberto Larcher, Marcia Ogasawara, Naike Casagrande, Marta Celegato, Cinzia Borghese, Zahid H. Siddik, Donatella Aldinucci, Anvarhusein A. Isab

**Affiliations:** ^1^ Center of Excellence in Nanotechnology (CENT), King Fahd University of Petroleum and Minerals, Dhahran, Saudi Arabia; ^2^ Department of Chemistry, King Fahd University of Petroleum and Minerals, Dhahran, Saudi Arabia; ^3^ Department of Translational Research, CRO Aviano National Cancer Institute, Aviano, PN, Italy; ^4^ Center for Technological Transfer, Edmund Mach Foundation, Trento, Italy; ^5^ The University of Texas MD Anderson Cancer Center, Department of Experimental Therapeutics, Houston, Texas, USA; ^6^ Department of Experimental Oncology 2, CRO Aviano National Cancer Institute, Aviano, PN, Italy

**Keywords:** gold(III) complexes, bipyridine, cisplatin resistance, reactive oxygen species, p53

## Abstract

We synthesized, characterized and tested in a panel of cancer cell lines, nine new bipyridine gold(III) dithiocarbamate-containing complexes. *In vitro* studies demonstrated that compounds 1, 2, 4, 5, 7 and 8 were the most cytotoxic in prostate, breast, ovarian cancer cell lines and in Hodgkin lymphoma cells with IC_50_ values lower than the reference drug cisplatin. The most active compound 1 was more active than cisplatin in ovarian (A2780cis and 2780CP-16) and breast cancer cisplatin-resistant cells. Compound 1 determined an alteration of the cellular redox homeostasis leading to increased ROS levels, a decrease in the mitochondrial membrane potential, cytochrome-c release from the mitochondria and activation of caspases 9 and 3. The ROS scavenger NAC suppressed ROS generation and rescued cells from damage. Compound 1 resulted more active in tumor cells than in normal human Mesenchymal stromal cells. Gold compounds were active independent of p53 status: exerted cytotoxic effects on a panel of non-small cell lung cancer cell lines with different p53 status and in the ovarian A2780 model where the p53 was knocked out. In conclusion, these promising results strongly indicate the need for further preclinical evaluation to test the clinical potential of these new gold(III) complexes.

## INTRODUCTION

The breakthrough of the anticancer properties of cisplatin *cis*-[Pt(NH_3_)_2_Cl_2_] around 1965, promoted very much attention in the area of metal-based anticancer agents [1]. The anticancer effects observed for cisplatin suggested that platinum and non-platinum metal-based compounds might be as valuable as organic anticancer drugs [2, 3]. Cisplatin, and a few related platinum complexes, such as carboplatin and oxaliplatin are along with the most commonly used anticancer agents [4]. The extensive clinical success of platinum compounds has improved the synthesis of other platinum and non-platinum metallodrugs that might demonstrate different cytotoxic properties and characterized by a different prototype of anticancer specificities, active against cisplatin-resistant tumor cells and with an encouraging toxicological profile. Thus, in the span of three to four decades, a variety of metal compounds were investigated as potential anticancer agents based on several non-platinum metals such as ruthenium, palladium, titanium, gold and copper [2–10].

It is known that Au(III) is iso-electronic with d^8^ system and iso-structural with Pt(II). Due to the structural similarity, the square planar gold(III) complexes have been qualified as appropriate candidates for the potential anti-cancer activity evaluation [3, 11–14].

Since the beginning of 21th century, a series of new gold(III) complexes turned out to be stable under physiological conditions and also exhibited appreciable *in vitro* and *in vivo* anticancer activity [12, 13, 15–18]. Parish et al. [19] described the synthesis of stable gold(III) complex using damp (2-(Dimethylamino)methyl)phenyl) as uni-negative bidentate ligand as an analogy in the structural features of cisplatin as neutral coordination complex in the mid of 1990s. The acceptable solution stability of these gold(III) compounds permitted to put into practice extensive both *in vitro* and in *in vivo* pharmacological testing. Therefore, several other series of cytotoxic gold(III) compounds were synthesized in other laboratories around the world, including the gold(III) dithiocarbamates [20] and the gold(III) porphyrins [21, 22].

Multidentate ligands such as polyamines, cyclam, terpyridine and phenanthroline were preferentially used in order to augment the stability of the gold(III) center and target gold(III) complexes with cationic coordination spheres [Au(en_2_)]^3+^, [Au(dien)Cl]^2+^, [Au(cyclam)]^3+^, [Au(phen)Cl_2_]^+^ and [Au(terpy)Cl]^2+^ were characterized. The investigation of their solution behavior was carried out through a variety of physical and chemical methods and demonstrated their stability [23, 24].

There are many examples of gold(III) complexes linked to dithiocarbamate, polyamine, polypyridine and their derivative ligands [2, 3]. In particular, dithiocarbamate ligands were combined with gold(III) complexes to inhibit the interaction of the metal center with thiol-containing renal enzymes and avoid cisplatin-induced nephrotoxicity [16].

In this connection, the idea of coupling the well-known anticancer properties of some metal ions with a potential chemo-protective function of dithiocarbamates was turned out to be an effective strategy. In fact, some gold(III) dithiocarbamate compounds were demonstrated to be cytotoxic, to inhibit both the proteasome and the thioredoxin reductase activity in human breast and prostate cancer cell cultures as well as in tumor xenografts [2, 3, 15, 17, 18, 25, 26].

Some other gold(III) complexes of bipyridine were synthesized. Akhmadullina et al. described the synthesis of [(bipy)AuCl_2_]BF_4_ and [(phen)AuCl_2_]BF_4_ [27]. Casini et al. prepared and characterized square planar gold(III) complexes that contain functionalized bipyridine ligands of general formula [Au(N^N)Cl_2_][PF_6_] [where N^N = 2,2′-bipyridine, 4,4′-dimethyl-2,2′-bipyridine, 4,4′-dimethoxy-2,2′-bipyridine and 4,4′-diamino-2,2′-bipyridine] [28].

Ogawa *et al*. [29] recently elaborated the introduction of a Au(III) ion into a mesogenic core, [M(Bdt)(Cnbpy)]^+^ (Bdt = 1,2-benzenedithiolato and Cnbpy = 4,4′-di-alkyl-2,2′-bipyridine (*n* = 13 (4,4′-di-tridecyl-2,2′-bipyridine (C1_3_bpy)) and 8,10 (4,4′-di-(3-octyltridecyl)-2,2′-bipyridine (C8,10bpy), leading to the formation of ionic molecular assemblies in crystalline and mesophases. Successive syntheses of precursor complexes, [AuCl_2_(Cnbpy)]PF_6_ (*n* = 13 (1) and 8,10 (2)), followed by the target complexes, [Au(Bdt)(Cnbpy)]PF_6_ (*n* = 13 (3) and 8,10 (4)), were achieved [29].

In order to improve the antitumor properties and reduce toxic side-effects of the previously reported gold(III) analogues, we designed new gold(III) complexes [Au(BPY)(S_2_CNR_2_)]Cl_2_ (BPY = 2,2′-bipyridine, 5,5′-dimethyl-2,2′-bipyridine, or 6,6′-dimethyl-2,2′-bipyridine and R = Methyl, Ethyl, or benzyl in S2CNR2 is Dialkyl/aryldithiocarbammate) with nitrogen and sulfur donor ligands. Here, we report synthesis, chemical characterization, and *in vitro* anticancer activity of these new gold(III) complexes.

## RESULTS

### Characterization by FT-IR

Gold(III) mixed ligands complexes 1–9 were identified *via* the presence of the stretching bands around 3030 and 2925 cm^−1^ for ν(C–H) the aromatic (phenyl and 2,2′-dipyridyl) and ν(C–H) saturated aliphatic methyl and ethyl groups of coordinated ligands respectively. All gold(III) compounds exhibited characteristic absorbance peaks primarily for ν(C–N) and ν(C–S). The infrared region 1480–1550 cm^−1^ is mostly related with the R_2_N–CSS ‘thioureide’ band in the IR spectra of dithiocarbamate compounds which defines the carbon-nitrogen bond order between a single bond at 1250–1350 cm^−1^ and a double bond at 1640–1690 cm^−1^ [30]. The thioureide band, ν(C–N) was detected at 1470–1490 cm^−1^ in compounds 1–9 respectively. These frequency absorption bands lie in between those associated with single C–N and double C = N bonds, hence the partial double bond character of ‘thioureide’ bond was confirmed for all gold(III) compounds [31]. The presence of strong absorption band in the range of 1470–1550 in FTIR spectra clearly indicates the formation dithiocarbamato gold(III) compounds [32, 33]. Similarly, C = S stretching with medium intensity around 1070 and 970 cm^−1^ for compounds 1–9 is an additional evidence of the formation of mixed ligands compounds. These absorption bands are comparable to free sodium salt of diethyldithiocarbamate ligands [31].

### Characterization by NMR

The ^1^H and ^13^C NMR chemical shifts of all nine compounds are given in synthesis part of experimental section and small downfield and upfield shifts for proton(s) of the coordinated dimethyl dithiocarbamate, diethyl dithiocarbamate and dibenzyldithiocarbamate have been seen in gold(III) compounds 1–9 in comparison to free dialkyl/diaryldithiocarbamate ligands [24, 34, 35]. The ^13^C NMR spectra of compounds 4, 7 and 1 showed seven and six resonances respectively; which confirmed the coordination of dimethyldithiocarmato and bipyridyl ligands with Au(III) ion (given in synthesis part of experimental section). The ^13^C NMR spectra of compounds 5, 8 and 2 showed one additional chemical shift comparative to compounds 4, 7 and 1 spectra as expected. Whereas, multiple chemical shifts are observed in the ^13^C NMR spectra of compounds 3, 6 and 9 due to presence of dibenzyl and pyridyl functional groups. There is an up-field chemical shift of NC = S carbon of coordinated dialkyl/diaryldithiocarbamate ligands with respect to free dialkyl/diaryldithiocarbamate ligands for all gold(III) compounds. The ^13^C chemical shifts of NC = S carbon of bonded dimethyl thiocarbamate, diethyl thiocarbamate and dibenzyl thiocarbamate are observed in the range 186–200 ppm in our synthesized compounds 1–9 [24, 34, 35].

### Mass spectrometry characterization

Positive-ion mode ESI mass spectra for electrosprayed aqueous-methanol solutions of compounds 1, 2 and 3 are presented in Supplementary Figure S1. The mass spectra profile of compounds 1, 2 and 3 present a common peak ion at 157 m/z corresponding to the protoned 2,2′-bipyridine moiety ligand (a) or its sodium adduct (b) at 179 m/z as reported for compound 3. The ESI-MS spectra of compound 1 presents an intense peak ion at 437 m/z assigned to a positive ion where Au(III) is coordinated by two dimethyl dithiocarbamate moieties. Compound 2 presents instead an intense peak at 493 m/z corresponding to the coordination of two diethyl dithiocarbamate moieties and analogously the ESI-MS scan for compound 3 presents a signal at 741 m/z associated to the coordination of two dibenzyldithiocarbamate moieties. Compounds 4, 5 and 6 in solution present the same ESI-MS behavior of compounds 1, 2 and 3 with the differences that in all ESI-MS spectra scan of compounds 4, 5 and 6 present a common positive Ion at 185 m/z that can be assigned to the free protoned dimethyl-2,2-bipyridine ligand (g) (Supplementary Figure S2). The structure of the dithiocarbamate derivatives c, d and f is shown in Supplementary Figure S3.

### Stability determination and interactions with lysozyme

Electronic spectra were recorded on each complex freshly prepared in DMSO solution at room temperature. Then, their electronic spectra were monitored over 24 hours at 37°C. The resulting UV-Vis absorption spectra of compound 1, 2 and 3 are shown in Supplementary Figure S4A–S4C. The UV spectra of the compounds confirm the stability under physiological temperature.

The interaction between compounds 1–3 and the lysozyme in solution have been probed by studying the change in their electrochemical profiles in presence of different concentration of protein (Supplementary Figures S5, S6 and S7). Both CV and SWV demonstrated that in the presence of lysozyme all compounds 1–3 showed a substantial decrease in the peak current as well as a clear peak potential shift (Supplementary Figures S5, S6 and S7). The significant change of their redox state due to their interaction with lysozyme suggests a significative chemical interaction of such gold compounds and proteins [36]. This chemical aspect deserves high attention because it could be involved in the mechanism of action of these innovative molecules as anticancer drugs.

### Cytotoxic effects

**Figure 1:**
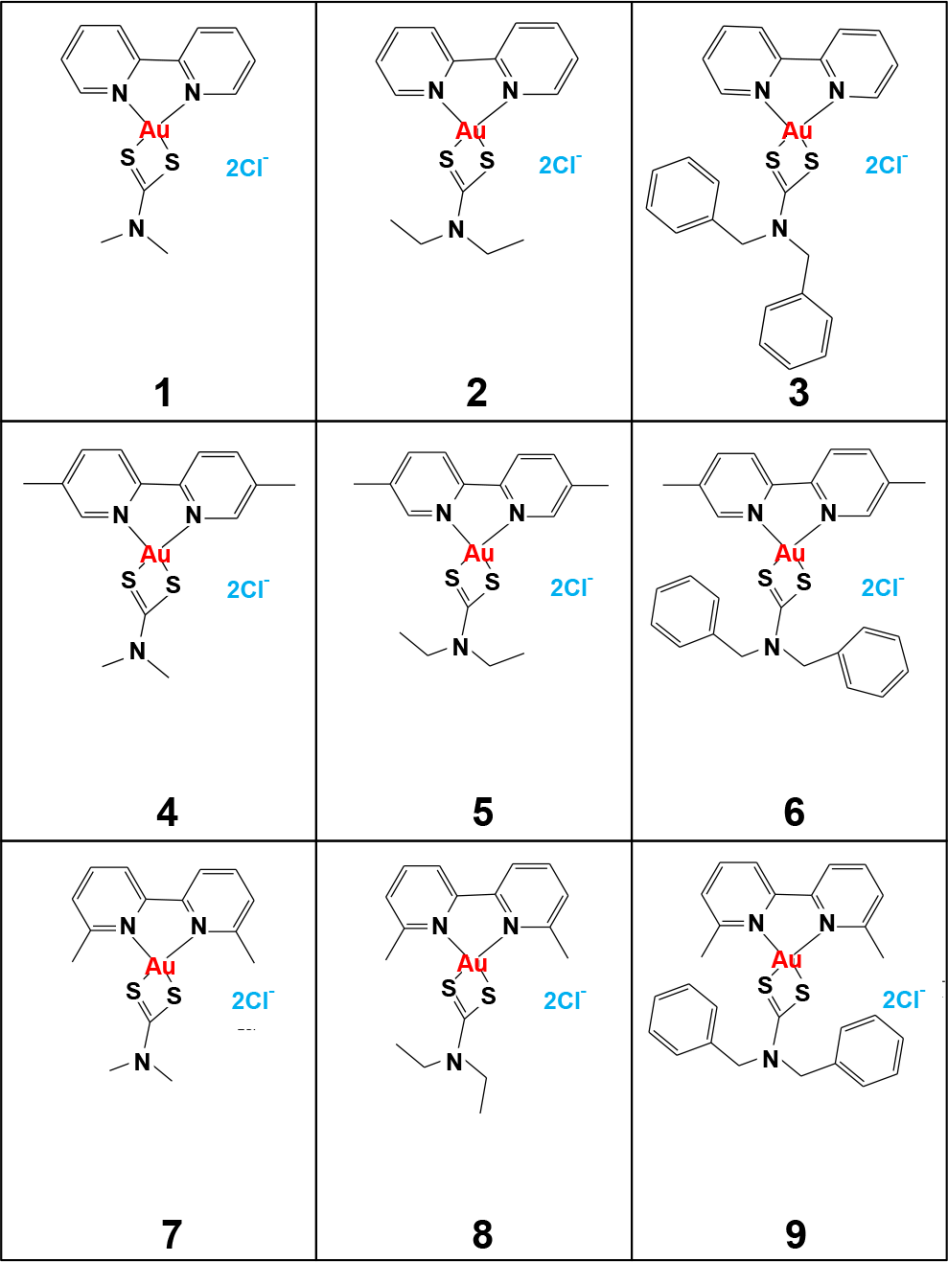
Chemical structure of the nine new synthesized gold(III) complexes (compound 1–9)

Gold(III)complexes (compounds 1–9) (Figure 1) were tested for *in vitro* cytotoxicity toward human Hodgkin lymphoma (L-540), androgen-resistant prostate cancer (PC3), breast cancer (MCF-7) and ovarian adenocarcinoma cisplatin-sensitive (A2780) and -resistant (A2780cis) cell lines. For comparison purposes, cisplatin activity was also evaluated.

**Figure 2:**
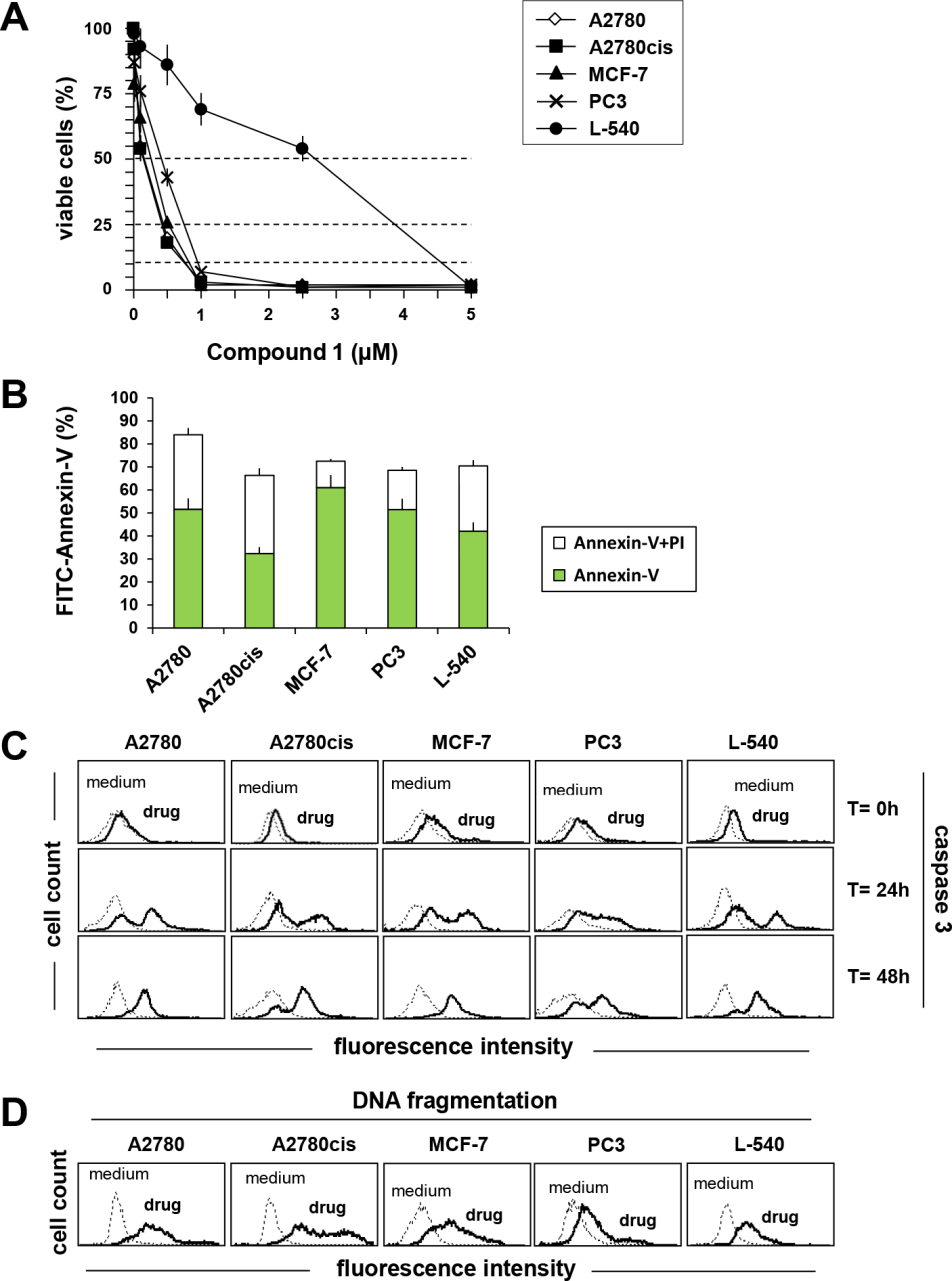
Compound 1 induces apoptosis in A2780, A2780cis, MCF-7, PC3, L-540 cells (**A**) Cells were exposed to increasing concentrations of compound 1. After 72 h the viable cell number was evaluated by MTT or MTS (**B**) FACS analysis of cells after 72 h incubation with compound 1 (IC_75_) and double stained with Annexin V/FITC and PI. (**C**) analysis of caspase 3 activation using FLICA reagent by flow cytometry after treating cells with compound 1 (IC_75_) for 0, 24 and 48 h. (**D**) DNA fragmentation (Apo-Direct) was assessed by flow cytometry after treatment for 72 h with compound 1 (IC_75_). Dotted lines indicate background fluorescence of cells. The x- and y-axes indicate the logarithms of the relative fluorescence intensity and relative cell number, respectively. FACS histograms are representative of one of three different experiments. Results represent the mean ± SEM of three independent experiments.

In L-540 cells gold(III) complexes showed an IC_50_ higher or similar to that of cisplatin (Table 1). Compounds 3, 6 and 9, all characterized by the addition of two benzoic groups (Figure 1, right panel), were less active than cisplatin in all cell lines tested showing higher or similar IC_50_ values (Tables 1 and 2). The other gold(III) complexes (compounds 1, 2, 4, 5, 7, 8) (Figure 1) were generally more active than the reference drug cisplatin with an IC_50_ ranging from 0.27 to 0.47 μM in MCF-7 cells, from 0.42 to 1.6 μM in PC3 cells (Table 1), from 0.18 to 0.73 μM in A2780 cells (Table 2) and from 0.17 to 0.45 μM in its cisplatin-resistant clone A2780cis (Table 2). Compound 1 showed the lowest IC_50_ values in the entire panel of the investigated tumor cell lines (Tables 1 and 2) and its representative dose-response curves are shown in Figure 2A. It was proved about 60-fold and 80-fold more active than cisplatin in inhibiting cell proliferation of cisplatin-resistant ovarian adenocarcinoma A2780cis cells (Table 2) and breast cancer MCF-7 cells (Table 1), respectively. With reference to Table 1, the IC_50_ values for complex 1, 2, 4, 5, 7 and 8 are comparable. Using three representative gold(III) complexes (compounds 1, 2 and 3) with different cytotoxic activity, we demonstrated the intracellular gold uptake by L-540 cells. Consistently with the cytotoxic effects, the gold uptake of both compound 1 and 2 was greater than that of compound 3 (Supplementary Figure S8A).

**Table 1:** Growth inhibition by gold(III) compounds in MCF-7, PC3 and L-540 cells

Compound		IC50 (μM)	
	MCF-7	PC3	L-540
**Cisplatin**	22.2 ± 0.20	3.30 ± 0.30	2.50 ± 0.10
**1**	0.27 ± 0.02	0.42 ± 0.04	2.68 ± 0.24
**2**	0.27 ± 0.02	0.70 ± 0.06	2.80 ± 0.25
**3**	23.0 ± 2.07	11.2 ± 1.00	12.0 ± 1.08
**4**	0.28 ± 0.02	0.59 ± 0.05	2.70 ± 0.22
**5**	0.29 ± 0.03	0.86 ± 0.08	2.90 ± 0.26
**6**	18.0 ± 1.62	11.6 ± 1.04	6.50 ± 0.06
**7**	0.35 ± 0.03	0.77 ± 0.07	3.60 ± 0.32
**8**	0.47 ± 0.04	1.60 ± 0.14	6.20 ± 0.56
**9**	31.0 ± 2.79	11.5 ± 1.03	35.75 ± 3.22

Cell lines were exposed to increasing concentrations of gold(III) compounds and Cisplatin included as reference drug. After 72 h exposure the number of viable cells was evaluated by MTT and MTS assay. Results represent the mean ± SEM of three replicate wells from three independent experiments. IC_50_ values were calculated using the Calcusyn software.

**Table 2:** Growth inhibition by gold(III) compounds in ovarian cancer cell lines A2780 and cisplatin-resistant A2780cis

Compound	IC_50_ (μM)		Fold resistance A2780cis/A2780ratio
	A2780	A2780cis	
**Cisplatin**	1.5 ± 0.1	10.4 ± 0.9	6.93
**1**	0.18 ± 0.02	0.17 ± 0.01	**0.94**
**2**	0.24 ± 0.02	0.18 ± 0.01	**0.58**
**3**	4.66 ± 0.13	14.3 ± 1.12	3.07
**4**	0.28 ± 0.03	0.17 ± 0.01	**0.94**
**5**	0.59 ± 0.05	0.23 ± 0.02	**0.39**
**6**	6.35 ± 0.06	15.7 ± 1.41	2.47
**7**	0.27 ± 0.02	0.23 ± 0.02	**0.85**
**8**	0.73 ± 0.06	0.45±0.03	**0.62**
**9**	6.7 ± 0.6	16.0 ± 1.44	2.39

Cell lines were exposed to increasing concentrations of gold(III) compounds and Cisplatin included as reference drug. After 72 h exposure viable cells were evaluated by MTT assay. Results represent the mean ± SEM of three replicate wells from three independent experiments. IC_50_ values were calculated using the Calcusyn software and compared with cisplatin. Compounds in which the ratio A2780cis IC_50_/A2780 IC_50_ resulted < 1 are reported as bold numbers.

### Induction of apoptosis

There are several mechanisms involved in the anticancer activity of Gold(I) and (III) complexes including the induction of apoptosis [15, 17, 37]. Therefore, we evaluated Annexin-V/PI double staining, caspase 3 activation and DNA fragmentation (Figure 2) in tumor cells treated with the most active gold(III) complex (compound 1). As shown in Figure 2B, compound 1 induced apoptosis in the cell lines tested. Treatment with compound 1 resulted in a substantial phosphatidylserine exposure (Annexin-V) together with an high percentage of cells permeable to PI staining, thus supporting apoptosis as a major mechanism of cell death (Figure 2B). Consistently, treatment with compound 1 (IC_75_) induced a time-dependent activation of caspase 3 (Figure 2C). Apoptosis induction was confirmed by DNA fragmentation (Figure 2D) with Apo-Direct analysis. Compound 1 was active at the same concentration in both cisplatin-sensitive (A2780) and -resistant (A2780cis) [38] ovarian cancer cells, and resulted more active in tumor cells than in normal human Mesenchymal stromal cells (MSCs) (Supplementary Figure S8B) (IC_50_ = 0.96 μM). The final DMSO concentration was un-effective (data not shown).

### Inhibition of mitochondrial functions and Reactive oxygen species (ROS) accumulation

**Figure 3:**
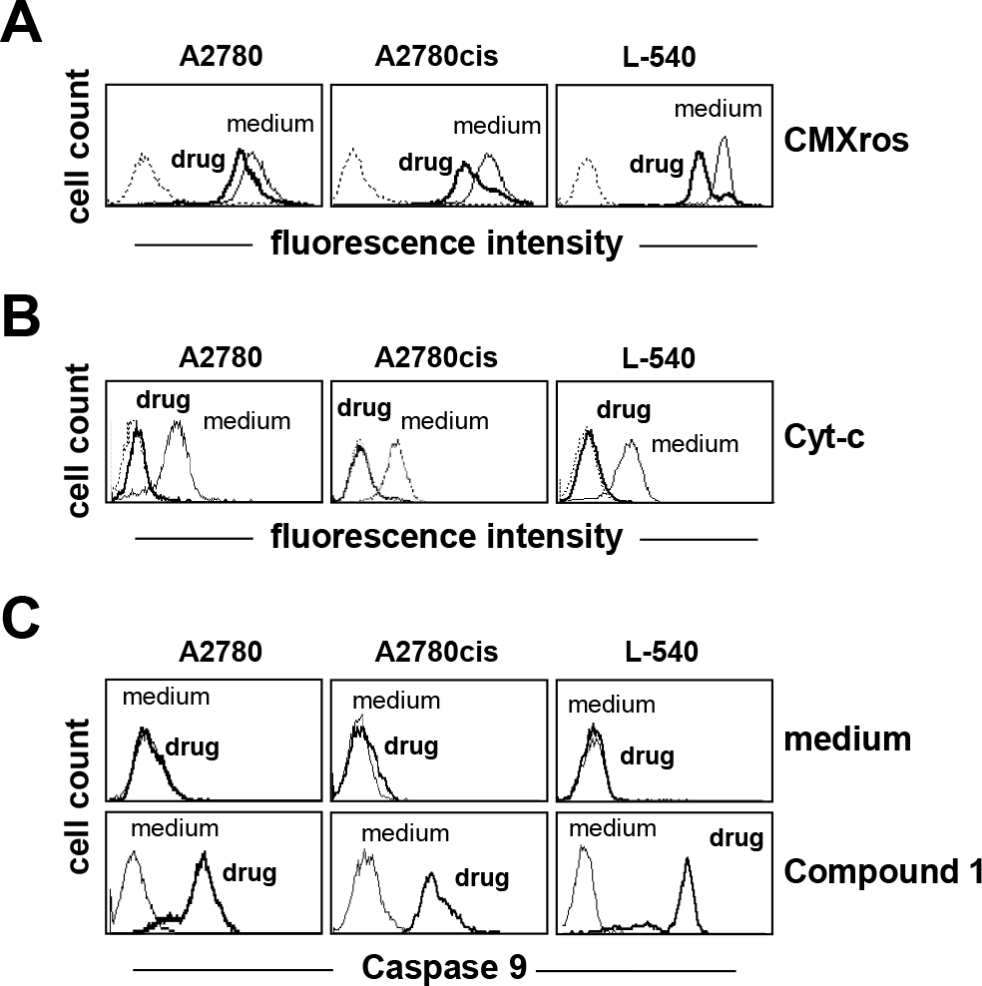
Compound 1 induces the mitochondrial intrinsic apoptotic pathway (**A**) mitochondrial membrane permeabilization (CMXRos), and (**B**) Cytochrome-c (Cyt-c) release were assessed by flow cytometry after treatment for 24 h with compound 1(IC_75_). (**C**) analysis of caspase 9 activation using FLICA reagent by flow cytometry after treating cells with compound 1 (IC_75_) for 24 h. Dotted lines indicate background fluorescence of cells. The x- and y-axes indicate the logarithms of the relative fluorescence intensity and relative cell number, respectively. FACS histograms are representative of one of three different experiment

**Figure 4:**
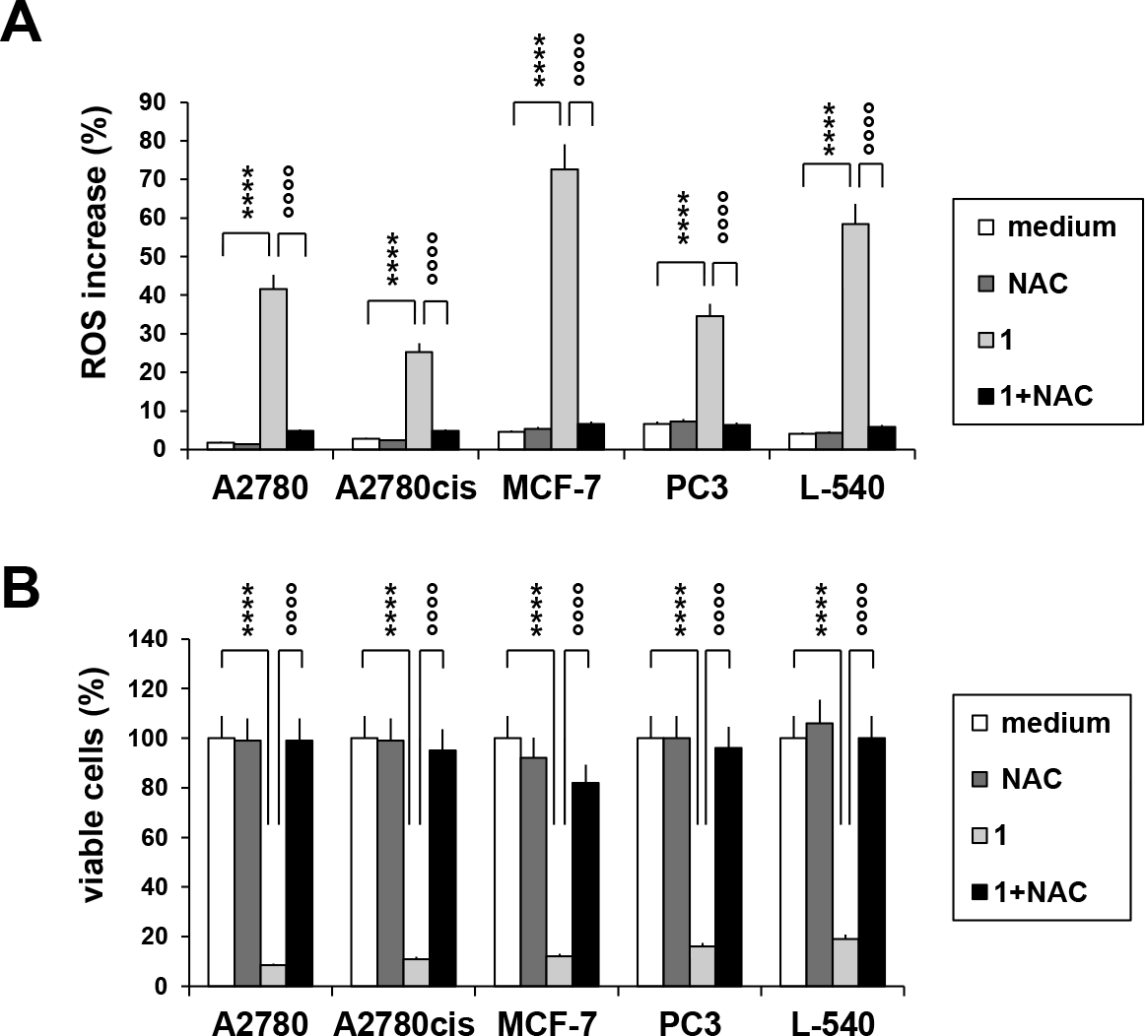
ROS accumulation after treatment with compound 1 (**A**) cells were treated for 72 h with compound 1 (IC_75_) in the presence or in the absence of the antioxidant NAC (5 mM), then stained with MitoSox reagent and analyzed by flow cytometry. Histograms represent the percentage of ROS positive cells. (**B**) percentage of cell viability upon treatment with compound 1 (IC_75_) and NAC (5 mM) for 72 h. Values represent the mean ± SEM of three different experiments. *p < 0.05; **p < 0.01; ****p < 0.0001, compound 1 *vs.* control; °°°°*p* < 0.0001, compound 1 *vs.* compound 1 + NAC.

Treatment with Compound 1 promoted mitochondrial membrane permeabilization (Figure 3A) and cytochrome-c (Cyt-c) release (Figure 3B) from the mitochondria and consistently induced activation of caspase 9 (Figure 3C), suggesting that the anticancer activity involves the mitochondrial intrinsic apoptotic pathway. Compound 1 (IC_75_) increased mitochondrial ROS levels, and especially in breast cancer MCF-7 cells (Figure 4A). In order to establish a possible relationship between ROS overproduction and decreased viability, tumor cells were incubated with the ROS scavenger NAC (Figure 4A and 4B). NAC blocked ROS production (Figure 4A) and almost completely neutralized the anti-proliferative effect of compound 1 (Figure 4B), suggesting that the cytotoxic/pro-apoptotic activity is due to ROS generation. Cell cycle progression was only slightly affected by compound 1 (data not shown).

### p53 status and Gold(III) complexes cytotoxicity

As response to chemotherapy can be influenced by the status of the tumor suppressor TP53 gene, four p53 wild-type (WT-p53), four p53 mutant (Mut-p53) and one p53 deleted (Null-p53) were utilized to determine whether the new gold(III) complexes have p53 dependence.

**Figure 5:**
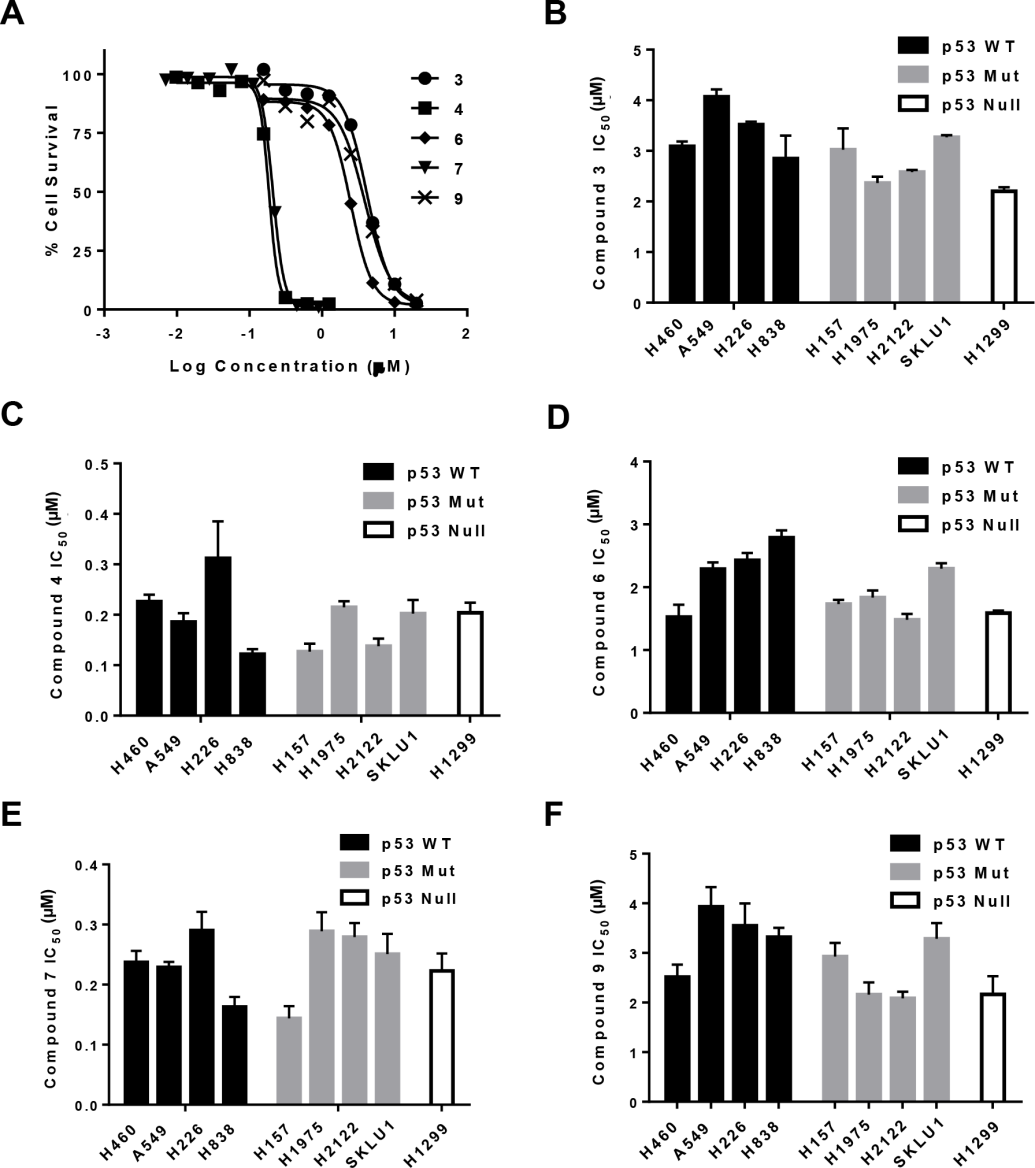
Cytotoxicity of gold complexes in a panel of NSCLC cell lines Determination of IC_50_ for compounds 3, 4, 6, 7 and 9 was by MTT assay. (**A**) Representative sigmoidal dose-response curves for A549 cells obtained 3 days after initiating exposure to the gold complex. (**B**–**F**), IC_50_ values to the indicated gold compounds for NSCLC cells grouped by p53 status. Data are presented as mean +/− SEM of three independent experiments.

**Figure 6:**
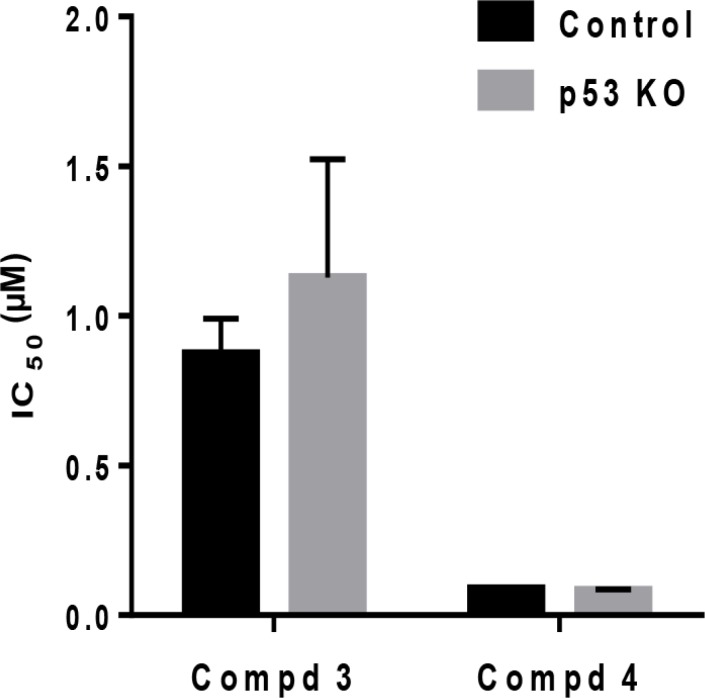
Cytotoxicity of gold complexes is independent of p53 The IC_50_ values for compound 3 and 4 were obtained in an A2780 clone following transfection with either CRISPR control (Control) or CRISPR p53-sgRNA plasmid (p53 KO). Data are presented as mean +/− SEM of three independent experiments.

Compounds (3, 4, 6, 7 and 9) showed an IC_50_ ranging between 0.16–4.1 μM. Representative dose-response curves in A549 cells are shown in Figure 5A, which indicate that compounds 4 and 7 have greater potencies than compounds 3, 6 and 9 also in lung cancer cells. Dose-response curves were used to obtain IC_50_ values in the entire NSCLC panel, and are shown in Figure 5B–5F. These IC_50_ values indeed demonstrate that compound 4 (IC_50_, 0.12–0.31 μM) and 7 (IC_50_, 0.14–0.29 μM) have greater potencies (at least 10-fold) than compound 3 (2.4–4.1 μM), 6 (1.5–2.8 μM) and 9 (2.1–3.9 μM) in all NSCLC cell lines. It is clear that structural differences regulate the potency of the molecules. Moreover, these results indicate that the IC_50_ values in WT- p53 models were similar to those in Mut- and Null-p53 cell lines. To confirm that sensitivity to gold(III) complexes was not dependent on p53 status, we utilized WT-p53 A2780 ovarian cancer cells in which p53 was knocked out using the CRISPR/Cas9 technology (CRISPR p53 KO). The corresponding control cells were transfected with a control plasmid and retained WT-p53 status. Results from the MTT assay revealed there were no significant differences in IC_50_ for representative compounds 3 (CRISPR Control, 0.87 ± 0.11 μM vs. CRISPR p53 KO, 1.13 ± 0.39 μM) and 4 (CRISPR Control, 0.087 ± 0.003 μM vs. CRISPR p53 KO, 0.083 ± 0.003 μM) (Figure 6).

Thus, the potential of the gold complexes to circumvent this resistance was investigated. For this purpose the IC_50_ and fold resistance (A2780cis/A2780) values for selected gold complexes were determined using the sensitive A2780 cancer cell line and the corresponding cisplatin-resistant A2780cis and 2780CP-16 [39] cell lines. While the A2780cis (resistant) cells showed an IC_50_ for cisplatin about 7-fold higher than A2780 (sensitive) cells, the IC_50_ of the most active compounds (1, 2, 4, 5, 7, 8) resulted similar or lower in A2780cis respect to A2780 (fold resistance or resistant factor less than 1) (Table 2). Similar results were obtained also in the 20-fold cisplatin-resistant 2780CP cells (data not shown), indicating that gold complexes under investigation in this study have the ability to overcome cisplatin-resistance to a substantial extent.

## DISCUSSION

The design of more active and less toxic antitumor agents is a major goal to develop new-targeted therapeutic strategies. Dithiocarbammate gold(III) complexes have recently gained increasing attention due to their strong cytotoxic effects associated with low nephrotoxicity and their capability to overcome cisplatin-resistance [15, 16].

In this study we described the synthesis and evaluated the anticancer activity of new bipyridine gold(III) dithiocarbamate-containing complexes. They demonstrated a potent cytotoxic activity in ovarian, lung, breast, prostate cancer and Hodgkin lymphoma cells, induced apoptosis and Reactive Oxygen species. They were active in cisplatin-resistant ovarian carcinoma cells and independently of p53 status in both lung and ovarian cancer cell lines and less toxic in non-cancer human Mesenchymal stromal cells.

These new gold(III) complexes have dithiocar­bamate ligands containing methyl (compounds 1, 4, 7) and ethyl (compounds 2, 5, 8) chains. The less active complexes (3, 6 and 9) have dithiocarbamate ligands containing dibenzyl substitution. The aliphatic containing dithiocarbamate ligands showed a higher anticancer activity respect to the benzyl dithiocarbamate complexes, summarized as Me ~ Et >> benzyl dithiocarbamate containing complexes. The different *in vitro* cytotoxic effects could be attributed to the small side chain of Methyl and Ethyl chains compared to the large dibenzyl group. However, even if our results indicate the consistent difference in potency (IC_50_) between different compounds, higher potencies are not an indicator of success at the clinical level, and this is well demonstrated by the ~10-fold difference in potency between cisplatin and the equal successful carboplatin [40]. Thus, other factors have to be taken into consideration to assess the clinical potential of less active new gold complexes, such as *in vivo* toxicity.

Cisplatin resistance is a critical factor that limits its clinical utility [1, 41]. The most active compounds (1, 2, 4, 5, 7, 8) were able to overcome the resistance to the reference drug cisplatin in ovarian and breast cancer cells, ruling out the occurrence of any cross-resistance phenomena. A fundamental mechanism of cisplatin resistance is the failure of this drug to activate p53, even in tumor cells primed for wild-type p53 function [42, 43]. Therefore, the identification of new drugs that do not depend for their anticancer activity on wild-type p53 status is an important goal since both NSCLC and ovarian cancer patients with p53 mutation have shorter overall survival as well as shorter time to progression [44, 45]. We found that the anticancer activity of the new gold(III) complexes was independent of p53 status: it was similar in WT-p53 models, in Mut- and Null-p53 cell lines and in WT-A2780 cells in which p53 was knocked out.

According to the above reported experimental results, complex 1 exerted a potent cytotoxic effect by inducing apoptosis, as assessed by Annexin-V, activation of caspase 3 and DNA fragmentation. Moreover, it induces mitochondrial membrane depolarization, cytochrome-c release and caspase 9 activation, indicating that its anticancer activity is exerted through the mitochondrial intrinsic apoptotic pathway. It induced ROS overproduction and growth inhibition, which was blocked by pre-treatment with the ROS scavenger NAC. However, we cannot exclude that thiol-containing antioxidant NAC could block the effects of compound 1 by binding with the active site of compound 1, as demonstrated for other gold complexes [46].

We found that the investigated compounds are able to interact with the small protein lysozymes. Moreover, the ESI-MS experiments indicated that in aqueous-methanol solution the compounds fast rearrange to generate stable derivatives where the gold(III) was coordinated by the four sulfur atom from two dithiocarbamate moieties. Indeed, we cannot exclude that such dithiocarbamate derivatives may have a role in the biological activity of the gold complexes. Further deep studies are however needed to better establish which chemical species is effectively responsible for the cellular cytotoxicity.

## MATERIALS AND METHODS

### Reagents

Disodium hydrogen phosphate, sodium dihydrogen phosphate, hen egg white lysozyme, and ethanol were obtained from Sigma-Aldrich. During the experimental work, the double distilled water was used for electrochemical measurements and acquired from Lab based Water Still Aquatron A 4000 D unit. Compounds (1–9) were dissolved (10 mM) in DMSO and stored at −80°C in volumes of 500 μL or aliquoted in volumes of 10 μL and stored at −20°C (used once without refreezing). They were then diluted in RPMI medium immediately before use. The culture medium with the same amount of drug-free DMSO was used as negative control in all experiments.

### Synthesis of gold(III) complexes

Sodium tetrachloroaurate(III) dihydrate, Sodium dimethyldithiocarbamate monohydrate, sodium diethyldithiocarbamate trihydarte, sodium dibenzyldithiocarbamate hydrate, 2,2′-Bipyridine, 5,5′-dimethyl-2,2′-bipyridine and 6,6′-dimethyl-2,2′-bipyridine were purchased from Sigma-Aldrich Co. St. Louis, Missouri United States. All solvents including ethanol, dichloromethane were purchased from Merck Darmstadt, Germany and used without further purification. All reactions were carried out under ambient conditions.

Elemental analyses of gold(III) complexes (compounds 1–9) were performed on Perkin Elmer Series 11 (CHNS/O), Analyzer 2400. The solid state FTIR spectra of free ligands and their corresponding gold(I) complexes were recorded on a Perkin–Elmer FTIR 180 spectrophotometer or NICOLET 6700 FTIR using Potassium bromide (KBr) pellets over the range 4000–400 cm-1. 1H and 13C NMR spectra were recorded on a LAMBDA 500 spectrophotometer operating at 500.01, 125.65 and 200.0 MHz respectively, corresponding to a magnetic field of 11.74 T. Tetramethylsilane (TMS) was used as an internal standard for 1H and 13C. The 13C NMR spectra were obtained with 1H broadband decoupling, and the spectral conditions were: 32 k data points, 0.967 s acquisition time, 1.00 s pulse delay and 45 pulse angle.

### [Au(BPY)(DMTC)]Cl_2_

The compound 1 was synthesized by stepwise synthesis; Na[AuCl_4_]. 2H_2_O, 0.5 mM (200 mg) and 2,2′-Bipyridine 0.5 mM (78 mg) were added simultaneously in 20 mL of ethanol and mixture was stirred for 3 h at room temperature. The sodium dimethyl dithiocarbamate dihydrate 0.5 mM (72 mg) in 10 mL distilled water was added slowly in the pale yellow turbid solution obtained of first step. The reaction mixture was stirred for an additional 1 h at room temperature. The final product appeared as light yellow precipitate in solution. The precipitate was collected by filtration, washed with fresh distilled water (3 × 10 mL) and dried at room temperature under vacuum. Yield: 83.09% (219.98 mg). FT-IR (KBr, υ_max_, cm^−1^): 3570 (m), 3045 (w), 2926 (w); 1586 (m), 1482 (s), 1242 (m), 1159 (w), 1039 (m), 993 (m), 967 (w), 761 (s), 565 (m), 439 (m). ^1^H NMR (500 MHz, DMSO-d_6_): δ = 3.36 (6H, 2 × CH_3_), 7.55, 8.06, 8.44 and 8.73 (2H, 2 × CH, BPY). ^13^C NMR (125.1 MHz, DMSO-d_6_): δ = 40.29 (CH_3_), 121.65, 125.19, 139.20 and 148.50 (2,2′-BPY), 189.87 (NC = S). Anal. calc. for C_13_H_14_Cl_2_N_3_S_2_Au (544.27): C, 28.69; H, 2.59; N, 7.72; S, 11.78%. Found: C, 28.55; H, 2.51; N, 7.75; S, 11.80%.

### [Au(BPY)(DETC)]Cl_2_

Compound 2 was synthesized according to the procedure as mention above for complex (1). The solution of sodium diethyldithiocarbamate trihydrate 1.0 mM (226 mg) in 10 mL of distilled water was added slowly to the reaction mixture of first step as described above for complex (1) and was stirred for 1 h at room temperature. The final product of compound 2 appeared as yellow precipitate in the reaction medium. The product was collected by filtration, washed with fresh distilled water (3 × 10 mL) and dried at room temperature under vacuum for 24 h. The product appeared as yellow crystalline powder. Yield: 86.87% (166.87 mg). FT-IR (KBr, υ_max_, cm^−1^): 3568 (m), 3048 (w), 2923 (w); 1583 (m), 1485 (s), 1247 (m), 1155 (w), 1038 (m), 988 (m), 958 (w), 768 (s), 555 (m), 435 (m). ^1^H NMR (500 MHz, DMSO-d_6_): δ = 1.25 (6H, 2 × CH_3_), 3.76 (4H, 2 × CH_2_), 7.60, 8.11, 8.49 and 8.76 (2H, 2 × CH, BPY). ^13^C NMR (125.1 MHz, DMSO-d_6_): δ = 12.07 (CH_3_), 46.57 (CH_2_), 121.63, 125.23, 139.23 and 148.36 (2,2′-BPY), 193.88 (NC = S). Anal. calc. for C_15_H_18_C_l2_N_3_S_2_Au (572.33): C, 31.48; H, 3.17; N, 7.34; S, 11.21%. Found: C, 31.37; H, 3.08; N, 7.19; S, 11.15%.

### [Au(BPY)(DBTC)]Cl_2_

Compound 3 was synthesized according to the procedure as mention above for compounds 1 and 2. The solution of sodium dibenzyldithiocarbamate hydrate 0.5 mM (148 mg) in 10 mL distilled water was added slowly in the reaction mixture of Na[AuCl_4_].2H_2_O, 0.5 mM (200 mg) and 2,2′-Bipyridine 0.5 mM (78 mg) as described above for complex (1) and stirred at room temperature for 1 h. The final product appeared as brown lumps in solution. The solid product was collected by filteration, washed with fresh distilled water (3 × 10 mL) and dried at room temperature under vacuum for 24 h. Yield: 85.55% (226.13 mg). FT-IR (KBr, υ_max_, cm^−1^): 3587 (m), 3143 (w), 2925 (w); 1552 (s), 1481 (m), 1224 (m), 1120 (w), 1078 (m), 981 (m), 916 (w), 551 (m), 473 (m). ^1^H NMR (500 MHz, DMSO-d_6_): δ = 5.03 (4H, 2 × CH_2_), 7.38 (10H, 2 × C_6_H_5_), 7.47, 7.96, 8.39 and 8.69 (2H, 2 × CH, BPY). ^13^C NMR (125.1 MHz, DMSO-d_6_): δ = 55.18 (CH_2_), 120.67, 124.43, 137.73 and 149.08 (2,2′-BPY), 128.24–132.50 and 154.64 (C_6_H_5_), 191.76 (NC = S). Anal. calc. for C_25_H_22_Cl_2_N_3_S_2_Au (696.46): C, 43.11; H, 3.18; N, 6.03; S, 9.21%. Found: C, 43.33; H, 3.15; N, 6.20; S, 9.33%.

### [Au(5,5′-Me_2_BPY)(DMTC)]Cl_2_

The compound 4 was synthesized by following the same procedure as described for compound 1 with minor modifications. The Na[AuCl_4_].2H_2_O, 0.5 mM (200 mg) and 5,5′-dimethyl-2,2′-dipyridyl 0.5 mM (92 mg) were added simultaneously in 20 mL of ethanol and mixture was stirred for 3 h at room temperature. The sodium dimethyldithiocarbamate dihydrate 0.5 mM (72 mg) in 10 mL distilled water was added slowly in the bright yellow turbid solution of above reaction mixture. The reaction mixture was stirred for 1 h at room temperature. The final product appeared as pale yellow precipitates in solution. The precipitates were collected by filtration, washed with fresh distilled water (3 × 10 mL) and dried at room temperature under vacuum. Yield: 80.09% (187.27 mg). FT-IR (KBr, υ_max_, cm^−1^): 3373 (m), 3037 (w), 2922 (w), 1555 (s), 1477 (s), 1272 (m), 1155 (m), 1028 (m), 981 (m), 896 (w), 567 (s), 468 (m). ^1^H NMR (500 MHz, DMSO-d_6_): δ = 2.37 (6H, 2 × CH_3_), 3.35 (6H, 2 × CH_3_), 7.85, 8.31 and 8.53 (2H, 2 × CH, BPY). ^13^C NMR (125.1 MHz, DMSO-d6): δ = 17.78 (CH_3_), 40.29 (CH_3_), 121.28, 135.33, 140.27 and 147.80 (2,2′-BPY), 193.87 (NC = S). Anal. calc. for C_15_H_18_C_l2_N_3_S_2_Au (572.33): C, 31.48; H, 3.17; N, 7.34; S, 11.21%. Found: C, 31.50; H, 3.08; N, 7.25; S, 11.17%.

### [Au(5,5′-Me_2_BPY)(DETC)]Cl_2_

The compound 5 was synthesized according to the procedure as mention above for compound 4. The solution of sodium diethyldithiocarbamate trihydrate 1.0 mM (226 mg) in 10 mL of distilled water was added slowly to the reaction mixture of first step as described above for compound 4 and was stirred for 1 h at room temperature. The final product appeared as deep yellow precipitate in the reaction medium. The product was collected by filtration, washed with fresh distilled water (3 × 10 mL) and dried at room temperature under vacuum for 24 h. The product appeared as yellow crystalline powder. Yield: 88.51% (343.9 g). FT-IR (KBr, υ_max_, cm^−1^): 3384 (w), 3035 (w), 2983 (w), 2926 (w), 1572 (s), 1473 (s), 1350 (m), 1220 (s), 1130 (m), 1056 (m), 996 (m), 827 (m), 538 (s), 465 (m). ^1^H NMR (500 MHz, DMSO-d_6_): δ = 1.27 (6H, 2 × CH_3_), 2.37 (6H, 2 × CH_3_), 3.76 (4H, 2 × CH_2_), 7.85, 8.31 and 8.53 (2H, 2 × CH, BPY). ^13^C NMR (125.1 MHz, DMSO-d_6_): δ = 12.05 (CH_3_), 17.80 (CH_3_), 46.55 (CH_2_), 120.41, 134.23, 138.83 and 148.64 (2,2′-BPY), 193.89 (NC = S). Anal. calc. for C_17_H_22_Cl_2_N_3_S_2_Au (600.38): C, 34.01; H, 3.69; N, 7.00; S, 10.68%. Found: C, 33.91; H, 3.64; N, 7.18; S, 10.59%.

### [Au(5,5′-Me_2_BPY)(DBTC)]Cl_2_

The compound 6 was synthesized according to the procedure as mention above for compound 4. The solution of sodium dibenzyldithiocarbamate hydrate 0.5 mM (148 mg) in 10 mL of distilled water was added slowly to the reaction mixture of first step as described above for compound 4 and was stirred for 1 h at room temperature. The final product appeared as yellowish green precipitates in solution. The product was collected by filtration, washed with fresh distilled water (3 × 10 mL) and dried at room temperature under vacuum for 24 h. Yield: 78.01% (256.6 mg). FT-IR (KBr, υ_max_, cm^−1^): 3388 (w), 3031 (w), 2993 (w), 2923 (m), 1553 (s), 1470 (s), 1345 (m), 1224 (s), 1123 (m), 1077 (m), 980 (s), 828 (m), 553 (s), 466 (m). ^1^H NMR (500 MHz, DMSO-d_6_): δ = 2.38 (6H, 2× CH_3_), 5.02 (4H, 2 × CH_2_), 7.37 (10H, 2 × C_6_H_5_), 7.90, 8.33 and 8.57 (2H, 2 × CH, BPY). ^13^C NMR (125.1 MHz, DMSO-d_6_): δ = 17.77 (CH3), 55.38 (CH_2_), 120.78, 134.69, 139.43 and 148.29 (2,2′-BPY), 128.25–132.52 and 150.13 (C_6_H_5_), 191.77 (NC = S). Anal. calc. for C_27_H_26_Cl_2_N_3_S_2_Au (724.52): C, 44.76; H, 3.62; N, 5.80; S, 8.85%. Found: C, 44.73; H, 3.70; N, 5.77; S, 8.90%.

### [Au(6,6′-Me_2_BPY)(DMTC)]Cl_2_

The compound 7 was synthesized by following the same procedure as described for compound 1 with minor modifications. The Na[AuCl_4_]. 2H2O, 0.5 mM (200 mg) and 6,6′-dimethyl-2,2′-dipyridyl 0.5 mM (92 mg) were added simultaneously in 20 mL of ethanol and mixture was stirred for 3 h at room temperature. The sodium dimethyldithiocarbamate dihydrate 0.5 mM (72 mg) in 10 mL distilled water was added slowly in the bright yellow solution of above reaction mixture. The reaction mixture was stirred for 1 h at room temperature. The final product appeared as light yellow precipitates in solution. The precipitates were collected by filtration, washed with fresh distilled water (3 × 10 mL) and dried at room temperature under vacuum. Yield: 90.09% (187.27 mg). FT-IR (KBr, υ_max_, cm^−1^): 3378 (m), 3033 (w), 2925 (w), 1575 (s), 1477 (s), 1238 (m), 1162 (m), 1046 (m), 998 (m), 868 (w), 567 (s), 440 (m). ^1^H NMR (500 MHz, DMSO-d_6_): δ = 2.49 (6H, 2 × CH_3_), 3.36 (6H, 2 × CH_3_), 7.41, 7.93 and 8.22 (2H, 2 × CH, BPY). ^13^C NMR (125.1 MHz, DMSO-d_6_): δ = 13.77 (CH_3_), 45.25 (CH_3_), 119.15, 125.21, 139.47 and 158.57 (2,2′-BPY), 187.83 (NC = S). Anal. calc. for C_15_H_18_Cl_2_N_3_S_2_Au (572.33): C, 31.48; H, 3.17; N, 7.34; S, 11.21%. Found: C, 31.67; H, 3.29; N, 7.38; S, 11.09%.

### [Au(6,6′-Me_2_BPY)(DETC)]Cl_2_

The compound 8 was synthesized according to the procedure as mention above for compound 7. The solution of sodium diethyldithiocarbamate trihydrate 1.0 mM (226 mg) in 10 mL of distilled water was added slowly to the reaction mixture of first step as described above for compound 4 and was stirred for 1 h at room temperature. The final product appeared as yellow precipitates in the reaction medium. The product was collected by filtration, washed with fresh distilled water (3 × 10 mL) and dried at room temperature under vacuum for 24 h. Yield: 68.51% (343.9 g). FT-IR (KBr, υ_max_, cm^−1^): 3418 (w), 3029 (w), 2978 (w), 2925 (w), 1574 (s), 1479 (s), 1351 (m), 1281 (s), 1154 (m), 1081 (m), 988 (m), 847 (m), 585 (s), 415 (m). ^1^H NMR (500 MHz, DMSO-d_6_): δ = 1.27 (6H, 2 × CH_3_), 2.49 (6H, 2 × CH_3_), 3.76 (4H 2 × CH_2_), 7.42, 7.92 and 8.23 (2H, 2 × CH, BPY). ^13^C NMR (125.1 MHz, DMSO-d_6_): δ = 13.07 (CH_3_), 24.96 (CH_3_), 47.71 (CH_2_), 119.50, 125.28, 139.52 and 158.55 (2,2′-BPY), 188.12 (NC = S). Anal. calc. for C_17_H_22_Cl_2_N_3_S_2_Au (600.38): C, 34.01; H, 3.69; N, 7.00; S, 10.68%. Found: C, 34.25; H, 3.51; N, 7.29; S, 10.48%.

### [Au(6,6′-Me_2_BPY)(DBTC)]Cl_2_

The compound 9 was synthesized according to the procedure as mention above for compound 7. The solution of sodium dibenzyldithiocarbamate hydrate 0.5 mM (148 mg) in 10 mL of distilled water was added slowly to the reaction mixture of first step as described above for compound 7 and was stirred for 1 h at room temperature. The final product appeared as reddish brown precipitates in solution. The product was collected by filtration, washed with fresh distilled water (3 × 10 mL) and dried at room temperature under vacuum for 24 h. Yield: 65.01% (256.6 mg). FT-IR (KBr, υ_max_, cm^−1^): 3428 (w), 3035 (w), 2978 (w), 2922 (m), 1566 (s), 1475 (s), 1331 (m), 1251 (s), 1119 (m), 1082 (m), 985 (s), 808 (m), 515 (s), 436 (m). ^1^H NMR (500 MHz, DMSO-d_6_): δ = 2.48 (6H, 2 × CH_3_), 5.01 (4H, 2 × CH_2_), 7.36 (10H, 2 × C_6_H_5_), 7.41, 7.91 and 8.20 (2H, 2 × CH, BPY). ^13^C NMR (125.1 MHz, DMSO-d_6_): δ = 24.92 (CH_3_), 54.88 (CH_2_), 118.95, 125.18, 138.33 and 157.43 (2,2′-BPY), 128.27–131.52 and 151.11 (C_6_H_5_), 199.01 (NC = S). Anal. calc. for C_27_H_26_Cl_2_N_3_S_2_Au (724.52): C, 44.76; H, 3.62; N, 5.80; S, 8.85%. Found: C, 44.33; H, 3.490; N, 5.63; S, 8.75%.

### Mass spectrometry characterization

Electro spray ionization mass spectrometry (ESI-MS) experiments for the 1–6 compounds were conducted using a 4000QTRAP Linear Ion Trap (ABsciex) mass spectrometer. Test solutions of 1 to 6 compounds in water /methanol (50%:50%) at concentration of 0.1 μg/mL were infused to Turbo V Ionspray TM ionization source of the mass spectrometer by a syringe pump Harvard apparatus (Holliston MA) and electrosprayed at a flow rate of 10 μL/min. The ESI source parameters use for ionization were: curtain gas CUR: = 20, declastering potential DP = 50, esi voltage: IS = 4000 V, source temperature TEM = 30°C entrance potential EP = :10 V and auxiliary gas G2 = 10 arbitrary unit. Positive ion mass spectra for each compound were acquired with MS-1 scanning from 100–1000 m/z using 2 sec time scan.

### Gold compound-protein interaction studies

The interactions of gold compounds with protein have been performed in solution by electrochemical investigations Voltammetry measurements were performed with an electrochemical workstation (CHI1140A, CH Instruments Inc., Austin, TX, USA). The Ag/AgCl reference electrode (in 3M KCl, CHI111, CH Instruments Inc), Glassy carbon working electrode (CHI 112, CH Instruments Inc) and platinum wire counter electrode (CHI115, CH Instruments Inc) inserted into the 5.0 ml glass cell. Due to the poor solubility of the present compounds in water, their solutions were prepared in ethanol. The GCE was polished as a mirror like surface with alumina slurry on the synthetic cloth before every electrochemical analysis. The cyclic voltammetry (CV) and square wave voltammetry (SWV) were scanned from −0.4 to 1.0 V for various analyses for compounds 1–3 in the absence and in the presence of different concentration of lysozyme under physiological environment.

### Cell lines and culture conditions

Hodgkin lymphoma (L-540) and human androgen-resistant prostate cancer (PC3) cell lines were obtained from the German Collection of Microorganisms and Cell Cultures (Braunschweig, Germany), human breast adenocarcinoma cell line MCF-7 (HTB-22TM) from the American Type Culture Collection (ATCC, Rockville, MD, USA). Human ovarian epithelial carcinoma-derived cancer cells A2780 and its cisplatin-resistant clone A2780cis were from Sigma Inc (St. Louis, MO,USA) and isogenic cisplatin-resistant 2780CP-16 model developed by sequential exposure of the A2780 cell line to increasing concentrations of cisplatin, as previously described [39]. Cisplatin was obtained from surplus clinical samples from the clinical pharmacy associated with Centro Riferimento Oncologico di Aviano. A panel of non-small cell lung cancer (NSCLC) cell lines H460, A549, H226, H838, H157, H1975, H2122, SKLU1 and H1299 were obtained from Dr. Garth Powis at MD Anderson Cancer Center. The parent cisplatin-resistant subclone (A2780cis) was maintained by weekly treatment with 1 μM cisplatin [38]. The resistance in 2780CP-16 cells was stable and did not require cisplatin maintenance. Cells were cultured at 37°C in 5% CO_2_ in RPMI (L-540, A2780, A2780cis, 2780CP-16, PC3 and the panel of lung cancer cells) or DMEM (MCF-7) medium supplemented with 10% heat-inactivated fetal calf serum (FCS; Sigma-Aldrich-Italy), 0.2 mg/ml penicillin/streptomycin and 0.1% (w/v) L-glutamine (Biocrom) at 37°C in a 5% CO_2_ fully humidified atmosphere.

Bone Marrow (BM) derived human Mesenchymal Stromal Cells (MSCs) were from Lonza (Lonza, Verviers, Belgium). MSCs were maintained in MSGM bullet kit (Lonza) and experiments were performed in DMEM medium (Cambrex Bio Science, Milano, Italy) supplemented with 10% FCS. To evaluate effects of compound 1, MSCs (5.0 × 10^3^) were seeded in 96-well flat-bottomed microplates (100 μL) and incubated for 24 h (to allow cell adhesion) before drug testing. The medium was then removed and replaced with fresh medium containing compound 1 to be tested at increasing concentrations (from 0.1 to 1 μM) at 37°C for 72 h. Each treatment was performed in triplicate. Cell proliferation was assayed using the MTT assay.

### Cellular uptake of gold compounds

L-540 cells (2.0 × 10^5^/mL) were seeded in 6 well plates and treated for 24 hrs with 0.5 μM of compounds 1, 2 and 3. After treatment cells were washed with ice-cold PBS four times, dry cell pellet digested with 700 μL of HNO3-HCl (1:3) solution for 2 h at 100°C and then resuspended in 4 mL of milliQ water. The gold analysis was performed with Agilent 7500 inductively-coupled-plasma mass spectrometer (ICP-MS). Results were expressed as ng/gold/10^6^ cells.

### Cell proliferation assay and p53 knockdown

PC3 and MCF-7 cells (2.5 × 10^**3**^), A2780, A2780cis (4.0 × 10^3^), A2780CP-16 and lung cancer cells (H460, A549, H226, H838, H157, H1975, H2122, SKLU1 and H1299) were seeded in 96-well flat-bottomed microplates (100 μL) and incubated for 24 h (to allow cell adhesion) before drug testing. The medium was then removed and replaced with fresh medium containing the gold(III) complexes (compound 1–9) to be tested at increasing concentrations (from 0.01 to 100 μM) at 37°C for 72 h. Each treatment was performed in triplicate. Cell proliferation was assayed using the MTT assay. Alternatively, L-540 cells (2.0 × 10^5^/mL) were seeded in 96-well plates and treated as previously described [37]. After treatment, cell proliferation was evaluated by the MTS assay (Promega). IC_50_ (i.e., the half maximal inhibitory concentration representing the concentration of a substance required for 50% *in vitro* inhibition) IC_75_ and IC_90_ values (Supplementary Table S1) were calculated using the CalcuSyn software (Biosoft, Ferguson, MO, USA) [47]. Dependence of activity on p53 was assessed in A2780 cells following p53 knockdown by CRISPR [48].

### Evaluation of apoptosis and ROS formation

PC3 (2.5 × 10^4^), MCF-7 (5.0 × 10^4^), A2780 and A2780cis (5.0 × 10^4^), and L-540 cells (2.0 × 10^5^/mL) were incubated in six-well plates with compound 1 (IC_75_) (Figure 1) for 72 h. Annexin-V binding (Becton-Dickinson [BD] Pharmingen, San Jose, CA) together with propidium iodide (PI) staining was detected in tumor cells by flow cytometry, as described [37, 38]. Caspase 3 and 9 activation was evaluated using the fluorochrome inhibitors of caspases (FLICA) Caspa-TagTM caspase-3/7 (FAM-DEVD-FMK) and caspase 9 (FAM-LETD) (Chemicon International, Milan, Italy). Briefly, cells were treated with compound 1 (IC_75_), then harvested, washed and resuspended in warmed complete medium supplemented with FLICA for 1 h and then immediately analyzed by flow cytometry. DNA fragmentation was assessed using the Apo-Direct kit (Becton-Dickinson Pharmigen, CA, USA) according to manufacturer's instructions. For mitochondrial ROS evaluation, cells treated with compound 1 were incubated with 5 μM of MitoSox reagent working solution (Molecular Probes, Invitrogen) for 30 minutes at 37°C. Red fluorescence was immediately analyzed by flow cytometry. Viable cells were identified according to their forward and right-angle scattering, electronically gated and analyzed on a FACScan flow cytometer (BD), using CellQuest software (BD). In another series of experiments, cells were exposed to compound 1 (IC_75_) in the presence or absence of the antioxidant and ROS scavenger N-acetyl-cysteine (NAC; 5 mM) (Sigma). After 72 h, ROS formation and viable cells number were evaluated by flow cytometry and trypan blue dye exclusion assay, respectively. To evaluate the dissipation of the mitochondrial membrane potential, 200 nM MitoTracker^®^ Red CMXRos (Molecular Probes, Invitrogen, Milan, Italy) was added to the cell culture for 30 min, then cells were washed twice and analyzed by flow cytometry. Cytochrome-c (Cyt-c) release was assessed using the mouse anti-Cytochrome-c antibody (BD), followed by PE-conjugated goat anti-mouse IgG (BD), as explained elsewhere [49].

## CONCLUSIONS

Drug resistance to chemotherapeutic agents is a central problem in oncology. Results in this investigation have identified members of a new class of gold(III) compounds as potential candidates for application in cancer chemotherapy. This is based on the demonstration that gold(III) compounds are effective in cisplatin-resistant, as well as in p53-defective cancers cells of different tumor types. Moreover, since our compounds contain a dithiocarbamate group capable of preventing a reaction with other sulphur-containing proteins, we may hypothesize a reduced toxicity *in vivo*. Thus, a gold(III) drug with an appropriate ligand, such as that presented in this study, has the potential to be an alternative to platinum-based drugs in cancer treatment. Further preclinical testing *in vitro* and *in vivo* will be essential to define their mechanism of action, their side effects (toxicity) and to select potential candidates for clinical activity.

## SUPPLEMENTARY MATERIALS


