# Supplementary material for: New bipyridine gold(III) dithiocarbamate-containing complexes exerted a potent anticancer activity against cisplatin-resistant cancer cells independent of p53 status
Source: Oncotarget. 2016 Nov 18;8(1):490–505. doi: 10.18632/oncotarget.13448 (PMC5341752; doi:10.18632/oncotarget.13448)
Supplement: Supplementary file 1 [file oncotarget-08-490-s001.pdf]

New bipyridine gold(III) dithiocarbamate-containing complexes exerted a potent anticancer activity against cisplatin-resistant cancer cells independent of p53 status

Supplementary Materials

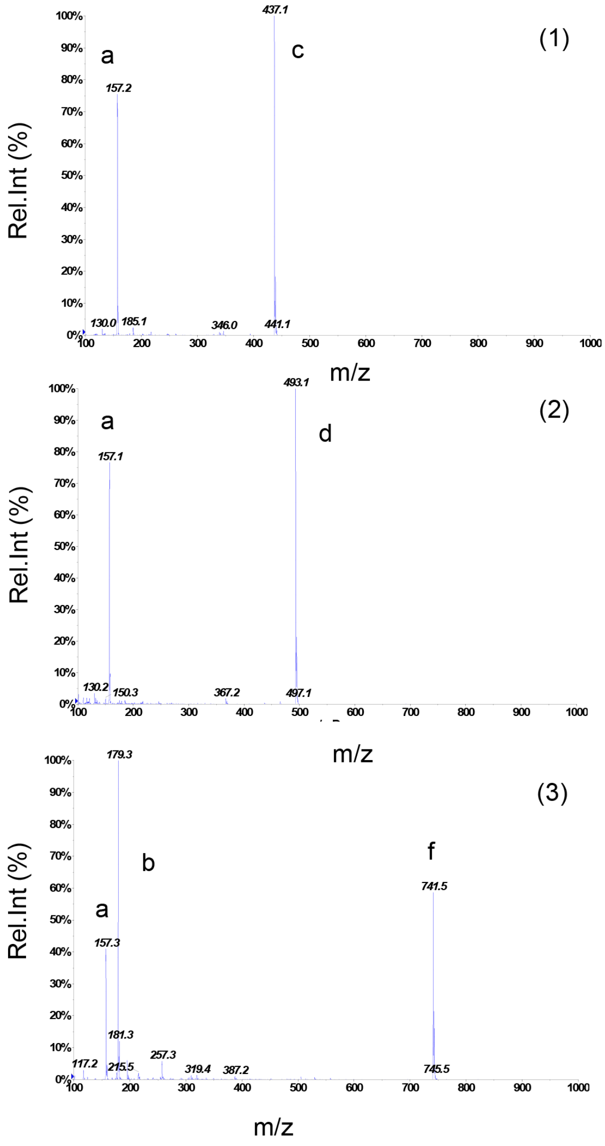

Supplementary Figure S1: Positive-ion mode ESI mass spectra for electrosprayed aqueous-methanol solutions of compound 1, 2 and 3.

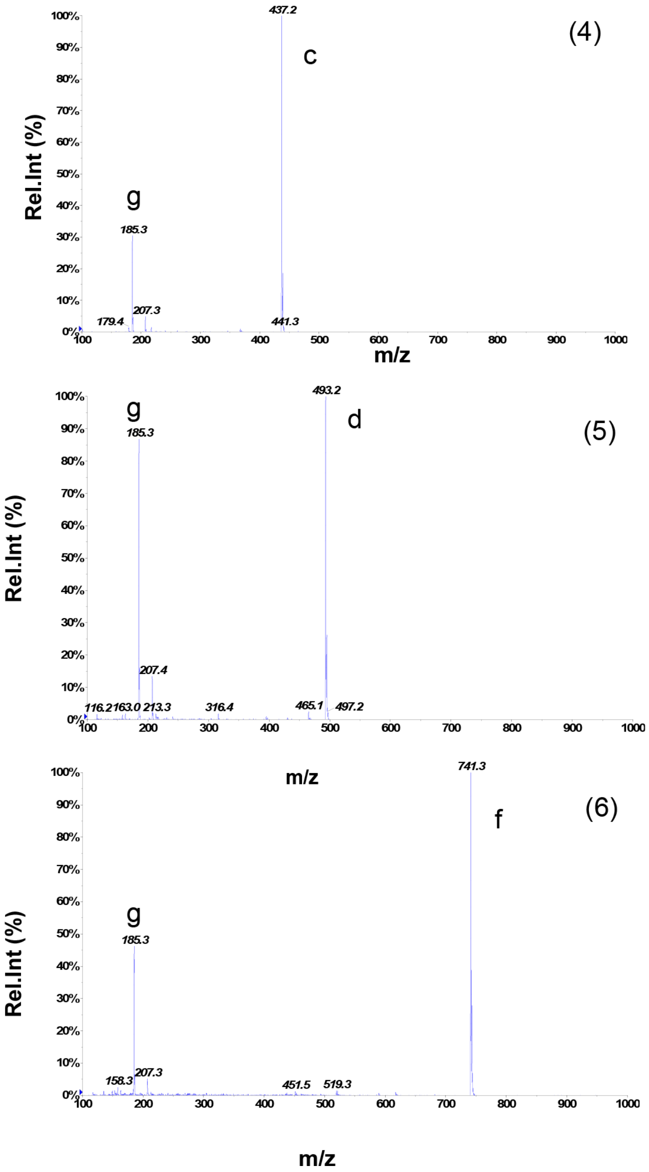

Supplementary Figure S2: Positive-ion mode ESI mass spectra for electrosprayed aqueous-methanol solutions of compound 4, 5 and 6.

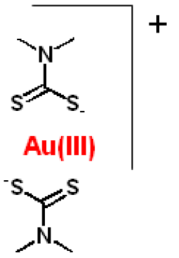

437.1 m/z

$[C_6H_{12}AuN_2S_4]^+$
Exact Mass: 436.95

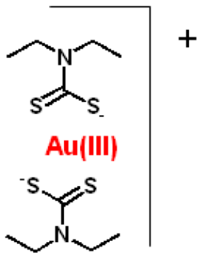

493.1 m/z

$[C_{10}H_{20}AuN_2S_4]^+$
Exact Mass: 493.02

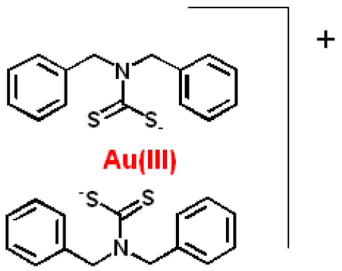

741.2 m/z

$[C_{30}H_{28}AuN_2S_4]^+$
Exact Mass: 741.08

Supplementary Figure S3: Structure of the dithiocarbamate derivatives c, d and f.

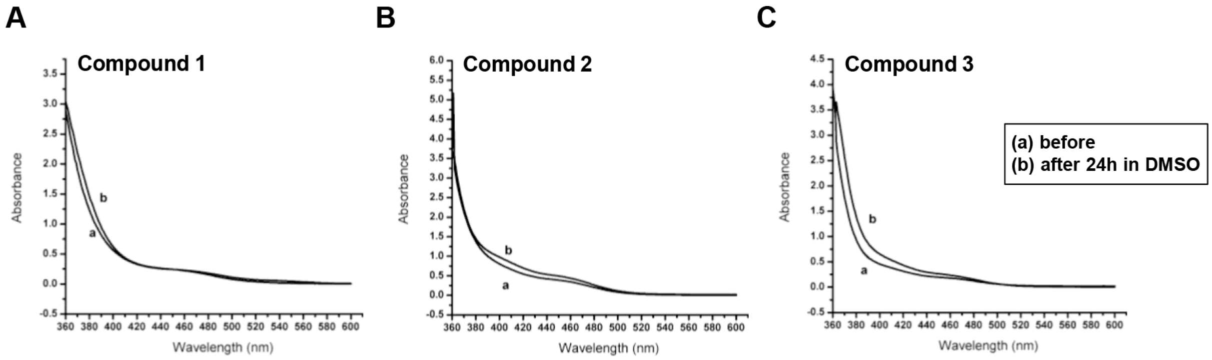

Supplementary Figure S4: UV-Vis absorption spectra of compound 1, 2 and 3.

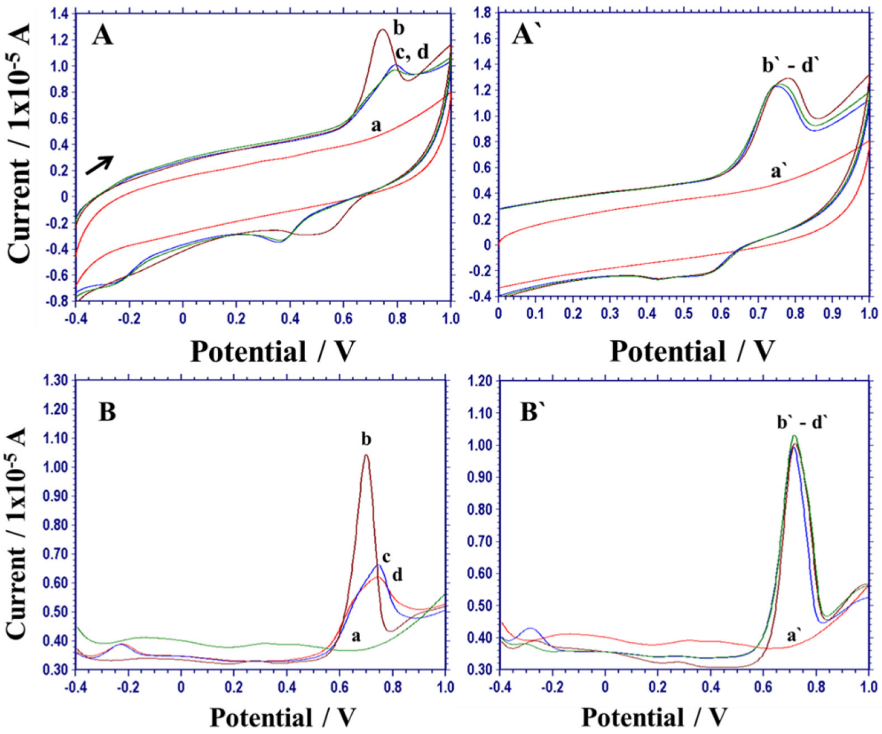

Supplementary Figure S5: (A) Cyclic voltammetry and (**B**) Square wave voltammetry of 0.5 mM of Compound 1 in PBS (pH 7.0) in absence (b) and presence of 10 μM (c) and 20 μM (d) of lysozyme. A` and B` are the corresponding control experiment. Accumulation potential, −0.2 V; accumulation time, 30 sec; pulse amplitude, 25 mV/sec; frequency, 15 Hz; potential increment, 4 mV.

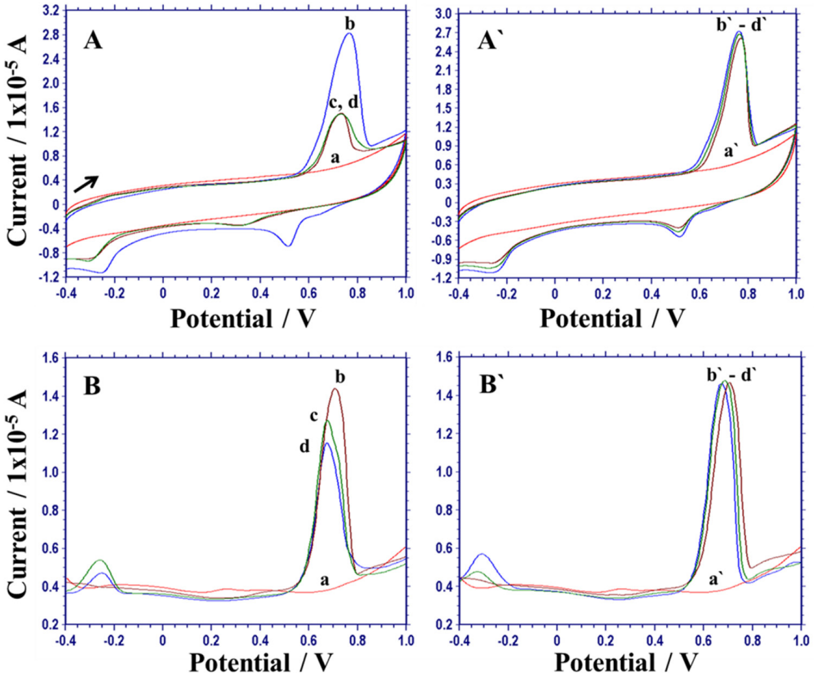

Supplementary Figure S6: (A) Cyclic voltammetry and (**B**) Square wave voltammetry of 0.5 mM of compound 2 in PBS (pH 7.0) in absence (b) and presence of 10 μM (c) and 20 μM (d) of lysozyme. A` and B` are the corresponding control experiment. Accumulation potential, −0.2 V; accumulation time, 30 sec; pulse amplitude, 25 mV/sec; frequency, 15 Hz; potential increment, 4 mV.

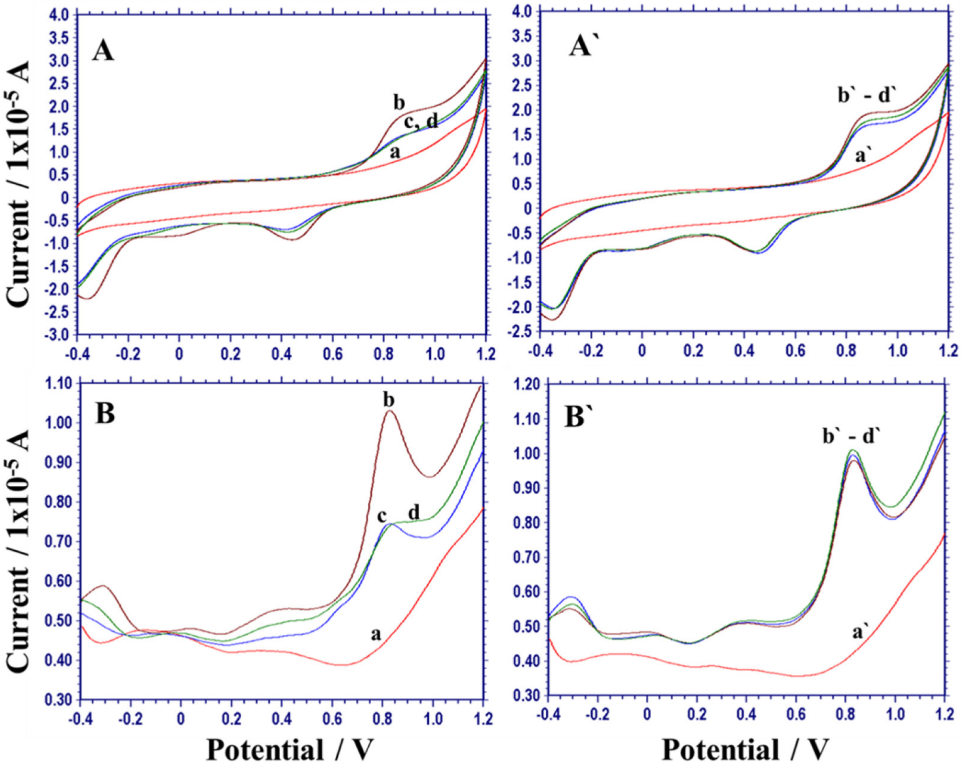

Supplementary Figure S7: (A) Cyclic voltammetry and (**B**) Square wave voltammetry of 0.5 mM of compound 3 in PBS (pH 7.0) in absence (b) and presence of 10 µM (c) and 20 µM (d) of lysozyme. A` and B` are the corresponding control experiment. Accumulation potential, −0.2 V; accumulation time, 30 sec; pulse amplitude, 25 mV/sec; frequency, 15 Hz; potential increment, 4 mV.

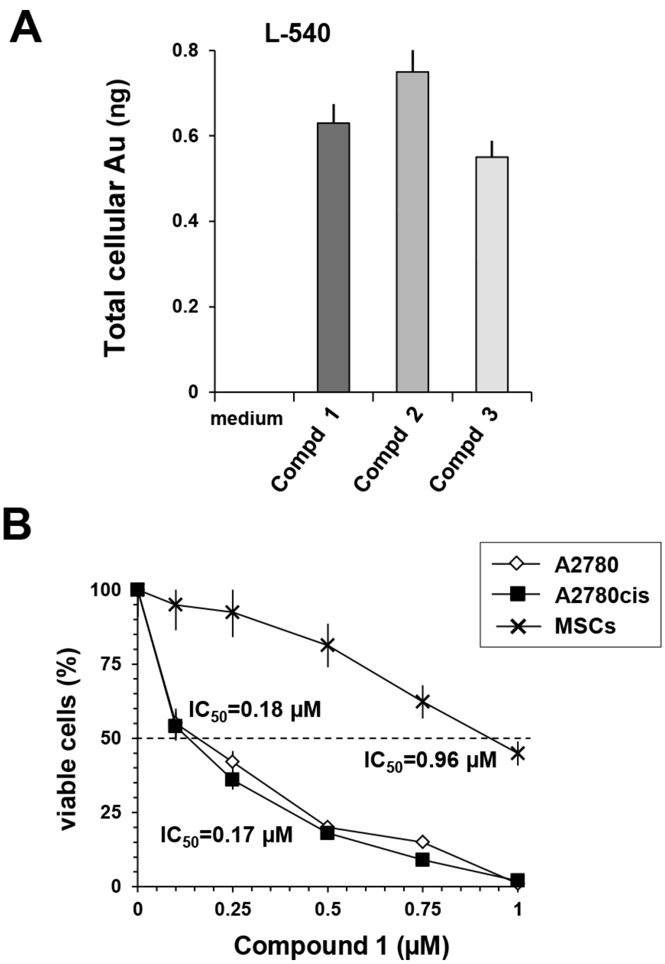

Supplementary Figure S8: (A) Gold complexes uptake by L-540 cells. L-540 cells were treated for 24 hrs with 0.5 µM of compounds 1, 2 and 3. Then gold complexes uptake was evaluated with ICP-Mass Spectrometer. The total celllular Au is expressed as mean ± SD from three independent experiments and it is referred to 10^6 cells .. (**B**) Compound 1 activity in human MSCs. MSCs, A2780 and A2780cis cells were cultured with compound 1. After 72 h, proliferation was evaluated by MTT assay. Results represent the mean ± SEM of three independent experiments.

Supplemental Table S1: Growth inhibition by gold (III) compound 1 in A2780, A2780cis, MCF-7, PC3 and L-540 cells

Compound 1	A2780	A2780cis	MCF-7	PC3	L-540
**IC_{50} (µM)**	0.18 ± 0.02	0.17 ± 0.01	0.27 ± 0.02	0.42 ± 0.04	2.68 ± 0.24
**IC_{75} (µM)**	0.45 ± 0.04	0.43 ± 0.04	0.51 ± 0.05	0.73 ± 0.07	3.50 ± 0.32
**IC_{90} (µM)**	0.60 ± 0.05	0.56 ± 0.06	0.74 ± 0.08	0.91 ± 0.11	4.20 ± 0.39

Cell lines were exposed to increasing concentrations of gold (III) compound 1, after 72 h exposure the number of viable cells was evaluated by MTT and MTS assay. Results represent the mean ± SEM of three replicate wells from three independent experiments. IC_{50} values were calculated using the Calcusyn software.
